# Representation of Ecological Systems within the Protected Areas Network of the Continental United States

**DOI:** 10.1371/journal.pone.0054689

**Published:** 2013-01-23

**Authors:** Jocelyn L. Aycrigg, Anne Davidson, Leona K. Svancara, Kevin J. Gergely, Alexa McKerrow, J. Michael Scott

**Affiliations:** 1 National Gap Analysis Program, Department of Fish and Wildlife Sciences, University of Idaho, Moscow, Idaho, United States of America; 2 Idaho Department of Fish and Game, Moscow, Idaho, United States of America; 3 United States Geological Survey Gap Analysis Program, Boise, Idaho, United States of America; 4 United States Geological Survey Gap Analysis Program, Raleigh, North Carolina, United States of America; 5 Department of Fish and Wildlife Sciences, University of Idaho, Moscow, Idaho, United States of America; University of Western Australia, Australia

## Abstract

If conservation of biodiversity is the goal, then the protected areas network of the continental US may be one of our best conservation tools for safeguarding ecological systems (i.e., vegetation communities). We evaluated representation of ecological systems in the current protected areas network and found insufficient representation at three vegetation community levels within lower elevations and moderate to high productivity soils. We used national-level data for ecological systems and a protected areas database to explore alternative ways we might be able to increase representation of ecological systems within the continental US. By following one or more of these alternatives it may be possible to increase the representation of ecological systems in the protected areas network both quantitatively (from 10% up to 39%) and geographically and come closer to meeting the suggested Convention on Biological Diversity target of 17% for terrestrial areas. We used the Landscape Conservation Cooperative framework for regional analysis and found that increased conservation on some private and public lands may be important to the conservation of ecological systems in Western US, while increased public-private partnerships may be important in the conservation of ecological systems in Eastern US. We have not assessed the pros and cons of following the national or regional alternatives, but rather present them as possibilities that may be considered and evaluated as decisions are made to increase the representation of ecological systems in the protected areas network across their range of ecological, geographical, and geophysical occurrence in the continental US into the future.

## Introduction

Traditionally, a mix of opportunity, available resources, and agency-specific conservation priorities are the foundation upon which networks of protected areas are developed over time [1]–[4]. This has led to a protected areas network in the continental US cultivated for multiple purposes including protecting biological resources, such as vegetation communities [5]–[8]. Often, to respond to conservation issues, such as habitat loss, the protected areas network is expanded by establishing new protected areas or enlarging existing ones [9]–[13]. However, with increasing land-use intensification the opportunities for expanding such networks are dwindling [4], [14]. Furthermore, with the imminence of climate change along with increased loss and fragmentation of vegetation communities, the exigency of protecting areas that represent the full suite of vegetation communities and therefore the species found therein, has increased [15]–[17].

The conservation community has increasingly focused on landscape levels for national decision making, but the lack of relevant and consistent data at a national scale has been an impediment [18]–[20]. Most public land management agencies, even those with the broadest authorities to protect natural resources have yet to implement ecosystem-scale approaches, perhaps due to lack of relevant data [21], [22]. However, the impediment that once prevented a national-scale approach to protected areas management in the continental US has recently been overcome with the availability of national-level data for vegetation communities, classified to ecological systems [23], and a protected areas database for the US [24]. Ecological systems are groups of vegetation communities that occur together within similar physical environments and are influenced by similar ecological processes (e.g., fire or flooding), substrates (e.g., peatlands), and environmental gradients (e.g., montane, alpine or subalpine zones) [23], [25]. Ecological systems represent vegetation communities with spatial scales of tens to thousands of hectares and temporal scales of 50–100 years. They represent the habitat upon which vertebrate species rely for survival. The Protected Areas Database of the US (PAD-US) represents public land ownership and conservation lands (e.g., federal and state lands), including privately protected areas that are voluntarily provided (e.g. The Nature Conservancy) [24]. Each land parcel within PAD-US is assigned a protection status that denotes both the intended level of biodiversity protection and indicates other natural, recreational and cultural uses (Table 1) [24]. Together, these databases provide the foundation for assessing the representation of vegetation communities in the continental US within the protected areas network and thereby informing decision making at the national level.

**Table 1:** Description of protection status categories in the Protected Areas Database for US [24].

Protection status	Description	Example
Lands managed to maintainbiodiversity (i.e., protected areasnetwork)	An area of land having permanent protection from conversion ofnatural land cover and a mandated management plan in operationto maintain a natural state within which disturbance events mayor may not be allowed to proceed without interference and/orbe mimicked through management.	Yellowstone National Park, Wyoming
Lands managed for multiple-use,including conservation	An area having permanent protection from conversion of naturalland cover for the majority of the area, but subject to extractive usesof either a broad low-intensity type (e.g., logging) or localized intensetype (e.g., mining). Protection of federally listed endangered andthreatened species throughout the area may be conferred.	Kaibab National Forest, Arizona
Lands with no permanent protectionfrom conversion, but may be managedfor conservation	An area with no known public or private institution mandates orlegally recognized easements or deed restrictions held by the managingentity to prevent conversion of natural habitats to anthropogenichabitat types. Conversion to unnatural land cover throughout isgenerally allowed and management intent is unknown.	Fort Irwin, California

Protection status denotes the intended level of biodiversity protection and indicates other natural, recreational, and cultural uses. These designations emphasize the managing entity rather than the land owner because the focus is on long-term management intent. Therefore an area gets a designation of permanently protected because that is the long-term management intent.

The protected areas network within the continental US is often viewed as one of our best conservation tools for securing vegetation communities and the species they support into the future [26]–[29]. An inherent assumption behind a network of protected areas is that protection of vegetation communities will also protect the species that rely on them, including invertebrate and vertebrate species, many of which little is known of their life history or habitat requirements [11], [30], [31]. For our analysis, we narrowly defined a protected area as an area of land having permanent protection from conversion of natural land cover and a mandated management plan in operation to maintain a natural state within which disturbance events may or may not be allowed to proceed without interference and/or be mimicked through management (Table 1) [24]. Furthermore, we defined a protected areas network as a system of protected areas that increase the effectiveness of *in situ* biodiversity conservation [32]. Lastly, we defined biodiversity as a hierarchy from genes to communities encompassing the interdependent structural, functional, and compositional aspects of nature [33].

The questions of how much of a vegetation community to protect and what approach is best for systematically protecting vegetation communities have been discussed at length [34], [35]. No single solution or specific amount of area has been established to meet both policy targets and biological conservation needs [35]. Most recently the Convention on Biological Diversity set a target of 17% for terrestrial areas in the Aichi Biodiversity Targets described within the Strategic Plan 2011–2020 [36]. The Aichi Biodiversity Targets also attempt to address biological needs by stating that areas protected should be ecologically representative [36]. Representation of vegetation communities is often put forth as a goal of conservation planning because the aim is to protect something of everything in order to conserve the evolutionary potential of the entire protected areas network [34], [37], [38]. The US has not explicitly addressed the representation of vegetation communities within the protected areas network; however, Canada has used representation targets to structure their protected areas network [39]–[41]. Even though climate change will likely alter what is represented within Canada’s protected areas network, starting from a representative group of protected vegetation communities provides a foundation for climate change adaptation [40], [41].

Numerous assessments of the US protected areas network and its effectiveness at conserving vegetation communities have all concluded the network is falling short [15], [20], [42]–[48]. Each assessment used the best data available at the time, but in all cases, extent, resolution, and consistency of the data were limited. Shelford [42] conducted the first assessment of protected areas in the US in 1926. His aim was to study the native biota of North America, which started with inventorying the existing protected areas and how their vegetation communities had been modified from pre-settlement conditions. Later, Scott et al. [15] found that 302 of 499 (∼60%) mapped vegetation communities within the US had <10% representation within protected areas. Dietz and Czech [20] found the median percentage of area protected within the continental US was 4% for the ecological analysis units they defined.

We recently have had the opportunity to evaluate the representation (i.e., saving some of everything) and redundancy (i.e., saving more than one of everything) of ecological systems within the existing protected areas network for the continental US. This opportunity was possible because of the availability of a complete ecological systems database for the continental US and a comprehensive database of the current protected areas network. Hence, we can now assess how well the protected areas network encompasses the ecological and evolutionary patterns and processes that maintain ecological systems and thereby the species that depend on them [37]. Additionally, based on the Aichi Biodiversity Targets within the Strategic Plan 2011–2020 of the Convention on Biological Diversity, we can evaluate the current protected areas network in the continental US in context of meeting the suggested 17% target for terrestrial areas [36].

If the current protected areas network is falling short of conserving vegetation communities then what potential alternatives might be available to address those shortfalls? One such alternative is to replace protected areas that contribute minimally to conservation of vegetation communities with those with greater conservation value [49]. The goal would be to increase the overall biodiversity protection of the entire protected areas network. This approach proposed by Fuller et al. [49] could be attractive because the sale of protected areas with less conservation value could go towards acquiring new ones. Fuller et al. [49] proposed this approach in Australia where a protected areas network has been systematically designed with broad representation of Australia’s vegetation types [49]. The protected areas network in the continental US has not been systematically designed [2], [4]. Would this approach be feasible if the criteria for determining the contribution to conservation (i.e., cost-effectiveness analysis) could be agreed upon consistently across the continental US?

Another alternative to address the current protected areas network’s shortfall could be to expand the network in area and number of protected areas [9], [11], [13]. A national assessment would be needed to identify vegetation communities not represented or under-represented within the existing protected areas network and a national conservation plan would be developed to prioritize acquisition of these vegetation communities to increase their representation on protected lands [50], [51]. There are approximately 300 million hectares of public and private lands with no permanent protection on which native vegetation communities occur [23], [24]. Could the representation of vegetation communities within the protected areas network be increased by prioritizing acquisition within these lands with no permanent protection?

A third alternative for addressing the protected areas network’s shortcomings might be to increase the emphasis of maintaining biodiversity on some public and private lands currently managed for multiple-use (Table 1). Swaty et al. [52] found that in addition to the 29% of the continental US land area that has been converted by human use; there were an additional 23% of non-converted lands with altered vegetation structure and composition, which likely are lands managed for multiple-use. The protected areas network is comprised of approximately 50 million hectares in the continental US, while there are about 140 million hectares of public and private lands managed for multiple-use [24]. Vegetation communities that are currently not represented or underrepresented within the current protected areas network may have representation on the approximately 140 million hectares of land managed for multiple-use [20], [24]. Could, therefore, an emphasis on maintaining biodiversity on a strategically targeted subset of lands managed for multiple-use be used to effectively expand the representation of vegetation communities within the entire protected areas network?

From a conservation management perspective for the US, the Department of Interior (DOI) has established a framework of Landscape Conservation Cooperatives (LCC) with the mission of landscape-level planning and management [53]. This national framework further supports the need for nationally consistent databases and analyses. We focused our analysis on alternative ways to potentially increase the representation of ecological systems in the protected areas network of the continental US. Specifically we asked (1) how well are ecological systems represented in the protected areas network relative to their occurrence in the continental US, including with regards to soil productivity and elevation, (2) how alternative approaches may potentially increase the representation of ecological systems in the protected areas network, and (3) how Landscape Conservation Cooperatives (LCC), the new landscape unit for conservation initiatives, can be used to regionally assess conservation status of ecological systems.

## Materials and Methods

### Data Description

We used the National Gap Analysis Program (GAP) Land Cover [23] and US Geological Survey GAP’s (USGS-GAP) Protected Areas Database of the US (PAD-US 1.0) [24] as the national datasets for our analyses. The land cover data contains 3 nested hierarchical levels of vegetation communities. Level I contains 8 groupings, based on generalized vegetative physiognomy (e.g., grassland, shrubland, forest), while Level II has 43 groupings representing general groups of ecological systems based on physiognomy and abiotic factors (e.g., lowland grassland and prairie, alpine sparse and barren). The third hierarchical level contains 551 map classes, including 518 ecological systems. We focused on the non-modified, non-aquatic classes at each level (Level I: 5 classes, Level II: 37 classes, and Level III: 518 ecological systems).

The National GAP Land Cover was compiled from the Southwest, Southeast, Northwest, and California GAP land cover data completed during 2004–2009 [23]. We incorporated data from LANDFIRE (www.landfire.gov) for the Midwest and Northeast. These national land cover data were based on consistent satellite imagery (Landsat Thematic Mapper (TM) and Enhanced Thematic Mapper (ETM)) acquired between 1999 and 2001 in conjunction with digital elevation model (DEM) derived datasets (e.g., elevation, landform) and a common classification system (i.e., ecological systems) to model natural and semi-natural vegetation [54]–[56]. The resolution is 30-m and typically the minimum mapping unit is 1 ha. Regional accuracy assessments and validations have been conducted and, based on those, in general, forest and some shrub ecological systems typically had higher accuracies than rare and small patch ecological systems, such as wetlands [57], [58].

PAD-US (Version 1.0) consists of federal, state, and voluntarily provided privately protected area boundaries and information including ownership, management, and protection status [24]. Protection status is assigned to denote the intended level of biodiversity protection and indicate other natural, recreational, and cultural uses (Table 1) [24]. In assigning protection status, the emphasis is on the managing entity rather than the owner and focuses on long-term management intent instead of short-term processes [11]. The criteria for assigning protection status includes perceived permanence of biodiversity protection, amount of area protected with a 5% allowance of total area for intensive human use, protection of single vs. multiple features, and the type of management and degree to which it is mandated [59]. The protection status ranges from lands managed to maintain biodiversity to lands with little or no biodiversity protection (Table 1). Lands managed for multiple-use, including conservation, are permanently protected, but allow for extractive uses, such as mining and logging. In the continental US, lands with no permanent protection are considered any land parcel not designated either of the other protection status categories. We included only lands permanently protected and managed to maintain biodiversity in our definition of the protected areas network.

We also used elevation data obtained from the National Elevation Dataset (NED) [60] and soil productivity. The National Elevation Dataset, a seamless dataset with a resolution of approximately 30 m, was the best available raster elevation data for the continental US [60]. We divided the National Elevation Dataset into 8 classes ranging from 0 to 4500 meters at 500-meter intervals. Soil productivity classes for the continental US were based on STATSGO data (http://soils.usda.gov/survey/geography/statsgo/). These data were reclassified into 8 soil productivity classes based on land capability classes (http://soils.usda.gov/technical/handbook) and ranged from very high to very low productivity.

To apply our analysis and results to current conservation management in the continental US, we used the LCC framework [53]. LCCs represent large area conservation-science partnerships between DOI and other federal agencies, states, tribes, non-governmental organizations (NGOs), universities, and other public and private stakeholders. Their intent is to inform resource management decisions to address landscape-level stressors, such as land use change, invasive species, and climate change [53].

### Data Analysis

The PAD-US 1.0 [24] and LCC data [53] were converted to grids (i.e., 30×30 m cells) and combined with the National GAP Land Cover [23] using ArcGIS 9.3.1 (ESRI, Redlands, CA). To assess the protection of ecological systems relative to their occurrence, we calculated a frequency distribution of protected area sizes within the existing protected areas network. To evaluate how the size range of protected areas would change with the inclusion of land managed for multiple-use, we calculated a frequency distribution of the protected areas network with lands managed for multiple-use added in (Table 1). We also calculated the amount of area of land managed for multiple-use needed to meet the 17% Aichi Biodiversity Target. To assess least protected or most endangered ecosystems, we summarized within each hierarchical level of the National GAP Land Cover (i.e., Levels I, II, and ecological systems) the number, size, protection status, and ownership of land parcels within PAD-US, as well as their distribution among LCCs. At the broadest level (Level I), we calculated percent availability versus percent protected to gain insight into the representation of each system in the protected areas network. We used a comparison index line (i.e., 1∶1 line) to indicate the relationship between percent availability and percent protected [61]. Similarly, we calculated the percent area of ecological systems protected (i.e., managed to maintain biodiversity), managed for multiple-use, and not permanently protected for soil productivity and elevation ranges by combining these data with PAD-US [24] using ERDAS Imagine 9.3 (Table 1).

The diversity of ecological systems across and redundancy within LCCs was calculated by counting the number of ecological systems occurring within each LCC. Diversity was defined as the number of ecological systems within each LCC, while redundancy was defined as the number of LCCs in which a single ecological system occurred [37]. For example, if an ecological system occurred in 2 LCCs, its redundancy value was 2. Unique ecological systems were those that occurred in a single LCC. Furthermore, we calculated the number and percent area protected of ecological systems by each protection status within each LCC. To assess whether lands were being protected at the same rate as those converted to human dominated classes, such as developed areas, cultivated croplands, orchards, vineyards, quarries, mines, gravel pits, oil wells, and pastures, we calculated the conservation risk index (CRI) for each LCC by dividing percent area converted by percent area managed to maintain biodiversity or percent area managed to maintain biodiversity and for multiple-use [23], [62]. Finally, we summarized CRI values by protection status.

## Results

**Figure 1:**
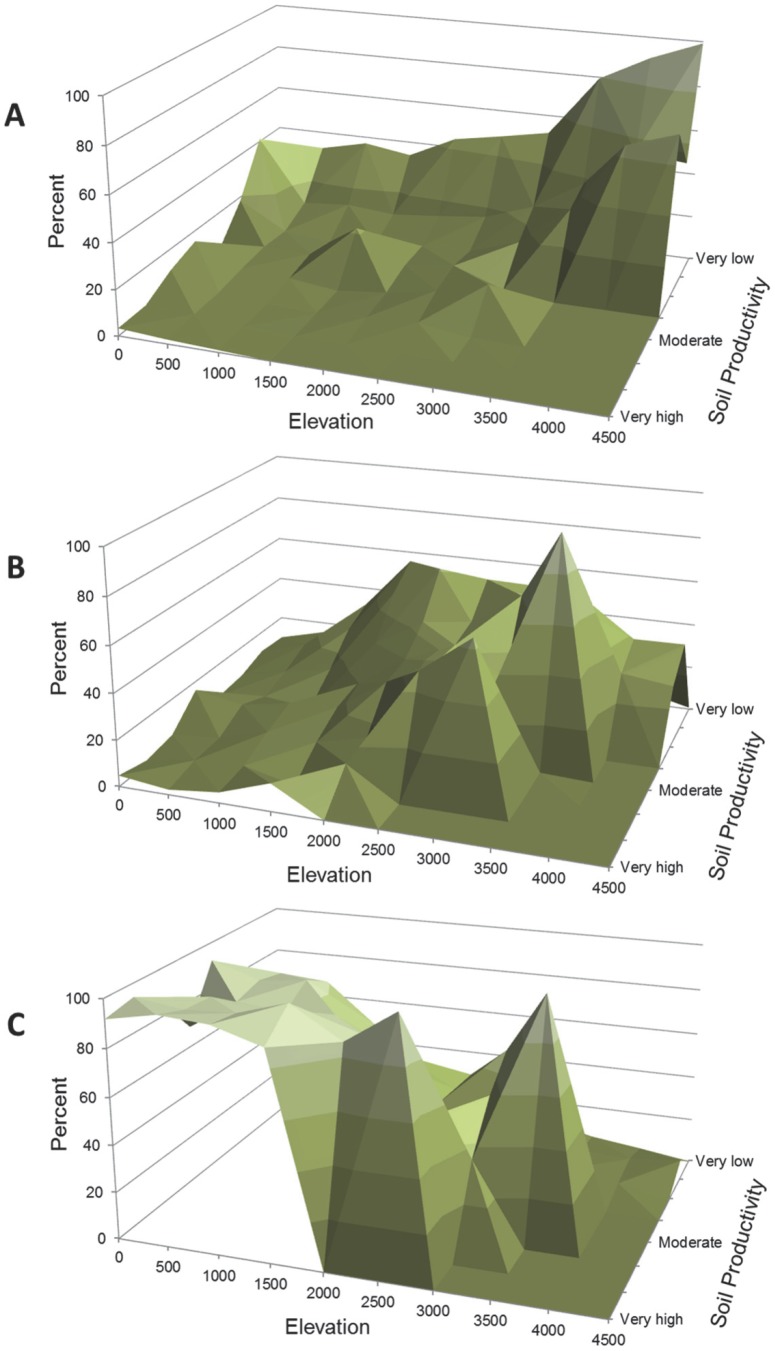
Percent area of ecological systems in relation to elevation, soil productivity, and protection status. Protection status designations include lands managed to maintain biodiversity (A), lands managed for multiple-use (B), and lands that have no permanent protection (C). See Table 1 for protection status descriptions. Percent area of ecological systems determined by combining data for elevation (meters) and soil productivity (http://soils.usda.gov/technical/handbook) with ecological systems grouped by protection status [23], [24], [60].

The current protected areas network in the continental US covers approximately 10% of the total area in which ecological systems occur. Across about 30,000 protected areas, the mean size of an individual protected area was 1942 ha with a size range of approximately 25–2,500,000 hectares over all protected areas. The analysis of representation of the network shows that the distribution of ecological systems managed to maintain biodiversity (i.e., the distribution of the protected areas network) is skewed towards high elevation and low productivity soils (Figure 1A). Overall 68% of all 518 ecological systems have <17% of their area protected, which is a target suggested by the Aichi Biodiversity Target of the Convention of Biological Diversity [36] and most of the ecological systems with <17% protected occur at low elevation and in areas with moderate to high productivity soils (Figures 1B and 1C, Table S1).

**Figure 2:**
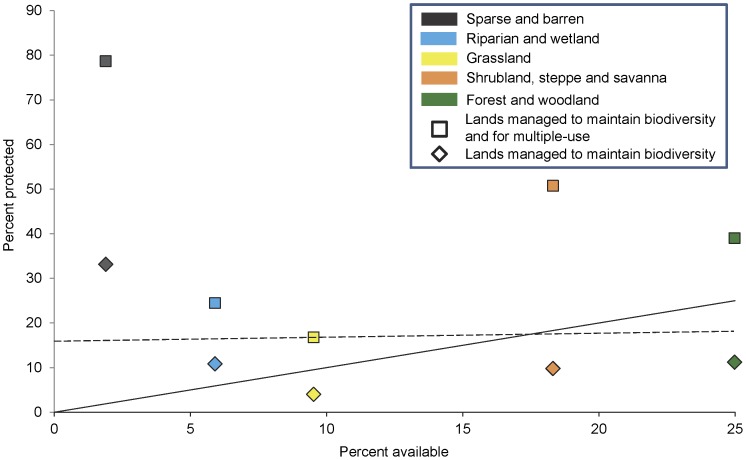
Percent protected and available for each Level I land cover group by protection status. Lands managed to maintain biodiversity (diamonds) are shown relative to lands managed to maintain biodiversity and for multiple-use (squares). See Table 1 for protection status descriptions. A comparison index line is shown, which indicates a 1∶1 relation between percent availability and percent protected [61]. A value below the 1∶1 line represents a Level I land cover group under-represented in the protected areas network, a value above represents a Level I land cover group well represented in the protected areas network, while a value on the line indicates a Level I land cover group available and protected equally [61]. For example, grassland, a Level I land cover group, has about 4% of its area managed to maintain biodiversity, but that increased to about 17% when lands managed for multiple-use were included [23], [24]. A dashed line representing the 17% Aichi Biodiversity Target of the Convention on Biological Diversity is shown [36].

**Figure 3:**
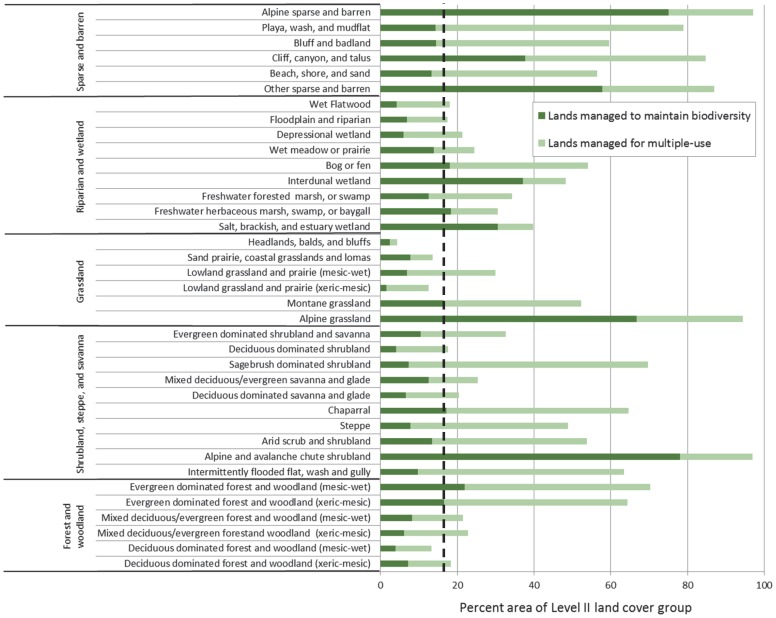
Percent area of Level II land cover groups by protection status. The Level II land cover groups are arranged by Level I land cover groups (see Table S1) [23]. Percent area for both lands managed to maintain biodiversity and lands managed for multiple-use are shown [24]. See Table 1 for protection status descriptions. A dashed line representing the 17% Aichi Biodiversity Target of the Convention on Biological Diversity is shown [36].

In examining the percent available versus percent protected for lands managed to maintain biodiversity, only two of the five Level I land cover groups (sparse and barren; riparian and wetland ) occurred above the 1∶1 line indicating a greater percentage of these groups are protected in relation to their availability (Figure 2). Representation of Level II land cover groups was lowest for lowland grassland and prairie (xeric-mesic), but most groups had <17% protected (Figure 3). Out of 37 Level II groups, 11 fell at or above the 17% Aichi Biodiversity Target [36].

Ecological systems on lands managed for multiple-use and on lands with no permanent protection comprised 29% and 61%, respectively, of the total area of the continental US in which ecological systems occur. When lands managed for multiple-use were included as part of the protected areas network, the overall number of protected areas increased to about 88,000 with a size range of approximately 25–117,757,000 hectares.

When both lands managed to maintain biodiversity and for multiple-use were included all five Level I land cover groups occurred above the 1∶1 line and all five occurred at or above the suggested 17% Aichi Biodiversity Target (Figure 2) [36]. The largest increases were within the shrubland, steppe, and savanna group, forest and woodland group, and sparse and barren group. The percent area of Level II land cover groups increased for all 37 groups when lands managed for multiple-use were added to lands managed to maintain biodiversity (Figure 3). The largest increases in percent area occurred within the lowland grassland and prairie (xeric-mesic) and sagebrush dominated shrubland. Out of 37 Level II groups, 33 fell at or above the 17% Aichi Biodiversity Target [36] when both lands managed to maintain biodiversity and multiple-use were included (Figure 3).

To meet the suggested 17% Aichi Biodiversity Target [36], approximately 9 million hectares (6.4%) of the 140 million hectares of public and private lands managed for multiple-use or 34 million hectares (11.3%) of the 300 million hectares of lands with no permanent protection would need to emphasize maintaining biodiversity or be acquired as part of the protected areas network (Table S1). Including lands managed for multiple-use with lands managed to maintain biodiversity, 98% of all ecological systems increased their percent area protected (Table S1). Using the suggested 17% Aichi Biodiversity Target [36], we found 32% of all ecological systems met that target, but that increased to 68% when lands managed for multiple-use were included (Table S1).

**Figure 4:**
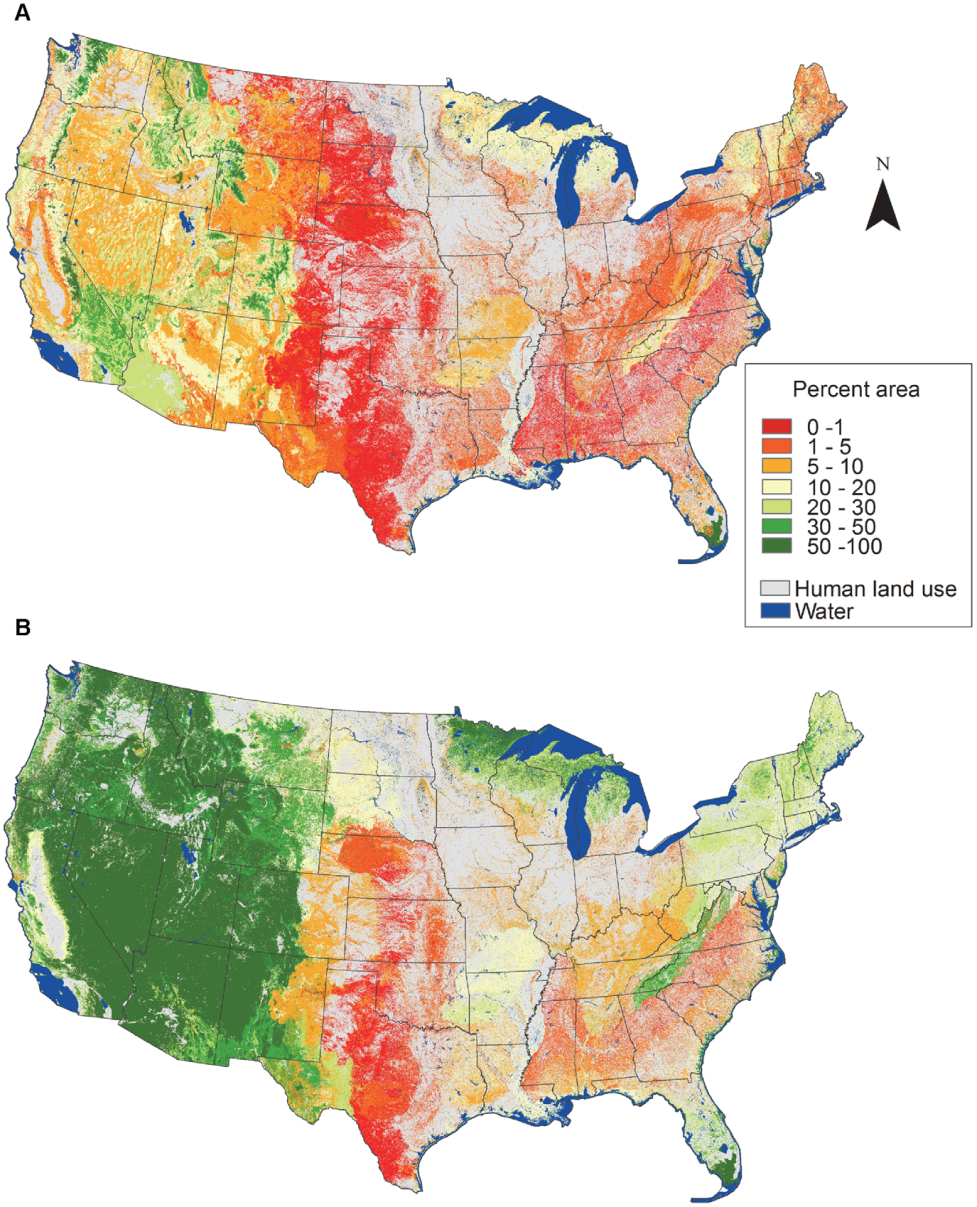
Percent area of ecological systems by protection status. Protection status designations are lands managed to maintain biodiversity (A) and lands managed to maintain biodiversity and multiple-use (B) for the continental US. Percent area is based on the area of each ecological system within each protection status divided by the total area of each ecological system [23], [24]. See Table 1 for protection status descriptions. Only non-modified, non-aquatic ecological systems were included (n = 518; Table S1).

**Figure 5:**
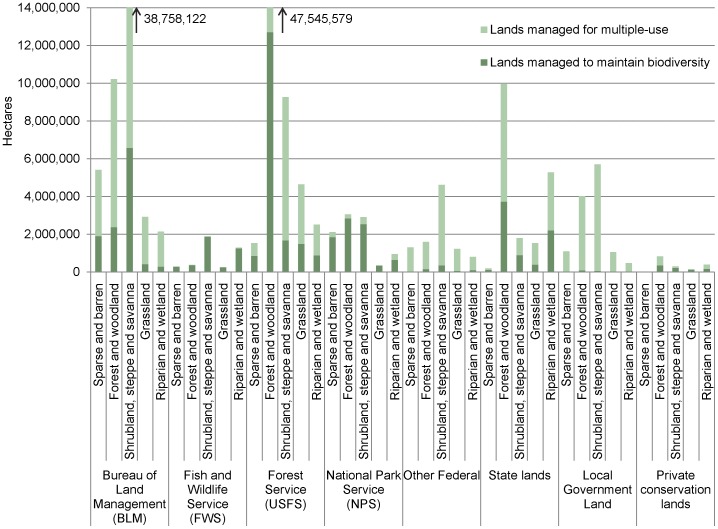
Area (ha) of Level I land cover groups by ownership and protection status. Ownership includes federal, state, and local governments as well as private conservation lands. See Table 1 for protection status descriptions. These values were for the continental US. Both BLM and USFS have areas of Level I land cover groups that fall outside the scale on this graph [23], [24]. Values for those Level I land cover groups are shown.

Including lands managed for multiple-use in the protected areas network would result in dramatic geographic changes in the western US, but noticeable changes were also evident in northeastern US, Florida, the Appalachian mountains, and around the Great Lakes (Figure 4). Federal, state, and local governments as well as private entities manage lands to maintain biodiversity and for multiple-use (Figure 5). There are approximately 50 million hectares of lands managed to maintain biodiversity with Bureau of Land Management (BLM) and US Forest Service (USFS) managing about 29 million hectares, which is more than US Fish and Wildlife Service (USFWS), National Park Service (NPS), and all other federal land combined (Figure 5). Approximately 140 million hectares is managed for multiple-use in the continental US with BLM and USFS managing about 100 million hectares (Figure 5, Table S1).

**Figure 6:**
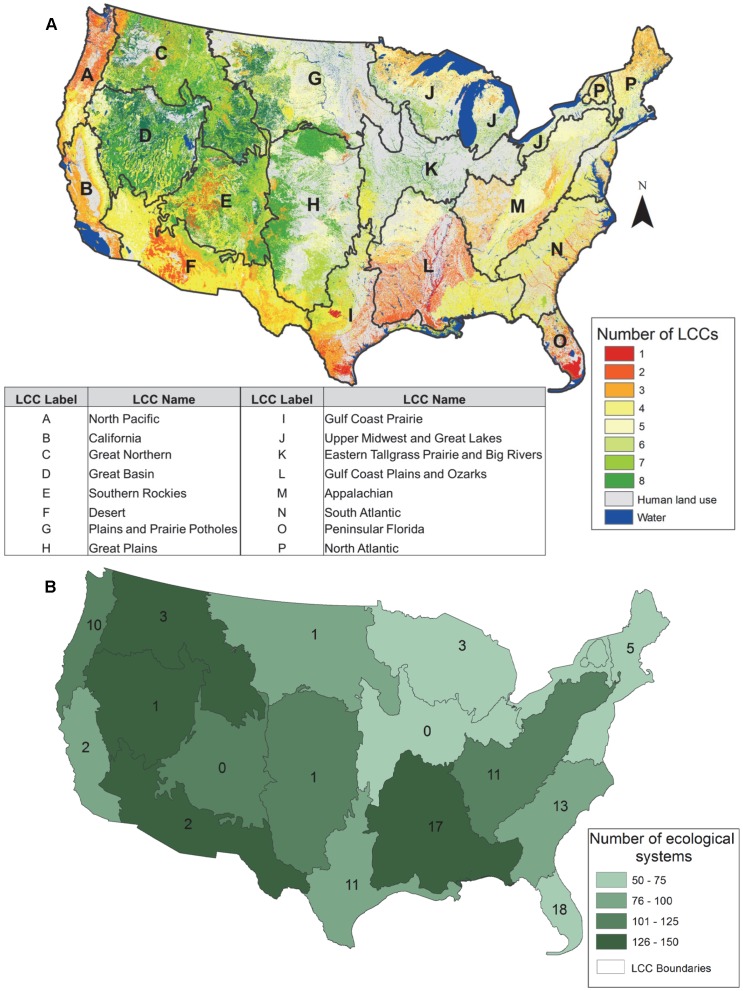
Redundancy, diversity, and uniqueness of ecological systems within Landscape Conservation Cooperatives (LCC). Redundancy measures the number of LCC’s in which a single ecological system occurs (A) [23]. The higher the number of LCC’s in which an ecological systems occurs the more redundancy displayed by that ecological system. For example, if an ecological system occurs in 2 LCCs, it has a redundancy value of 2. Diversity is the total number of ecological systems occurring with an LCC, which is shown by color shading of LCCs (B). Uniqueness is the number of ecological systems that occur in a single LCC, which is indicated by the number within each LCC (B). For example, the Great Northern LCC encompasses 126–150 ecological systems total, most of these occur in a total of 7 or 8 LCCs, but 3 are unique and only found in this LCC. Only non-modified, non-aquatic ecological systems were included (n = 518; Table S1). Each LCC is assigned a letter, which indicates the name of the LCC.

**Figure 7:**
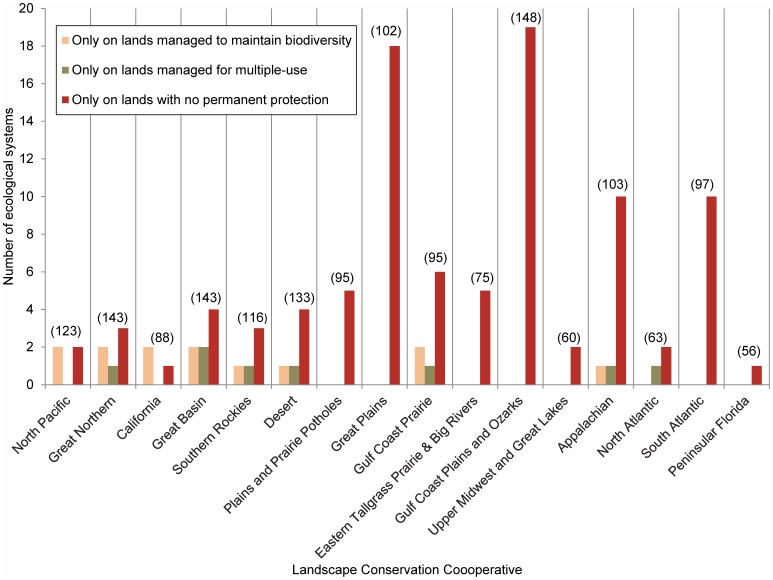
Number of ecological systems occurring only within each protection status by Landscape Conservation Cooperative (LCC). Ecological systems included occur only within the specified protection status [23], [24]. The total number of ecological systems within each LCC is shown parenthetically. For example, the Great Plains LCC contains 102 ecological systems with 18 occurring only on lands with no permanent protection and none occurring on lands managed to maintain biodiversity or for multiple-use. See Table 1 for protection status descriptions. Only non-modified, non-aquatic ecological systems are included (n = 518; Table S1).

**Figure 8:**
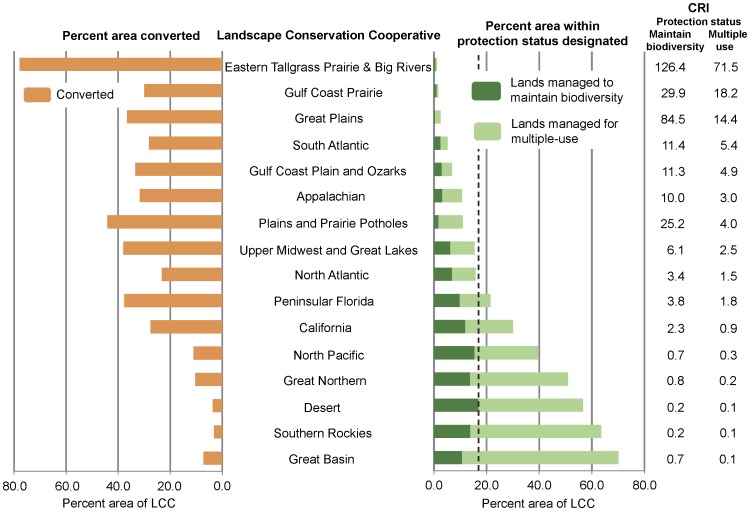
Percent area of Landscape Conservation Cooperative (LCC) protected or converted and its conversion risk index (CRI). CRI for each LCC is calculated by dividing percent area converted by percent area protected [62]. The CRI index is shown for lands managed to maintain biodiversity (i.e., labeled maintain biodiversity) as well as for lands managed to maintain biodiversity and multiple-use (i.e., labeled multiple-use) [23]. The LCCs are ordered by percent area within each protection status. See Table 1 for protection status descriptions. A dashed line representing the 17% Aichi Biodiversity Target of the Convention on Biological Diversity is shown [36].

Redundancy values for ecological systems occurring in LCCs ranged from 1–8, with redundancy values higher in LCCs in the west (Figure 6A). Ecological systems were highly diverse in 4 LCCs (Great Northern, Great Basin, Desert, and Gulf Coast Plain and Ozarks); however, only 1 had numerous unique ecological systems (Gulf Coast Plains and Ozarks; Figure 6B and Table 2). When including lands managed for multiple-use in the protected areas network, 7 out of the 16 LCCs in the continental US more than doubled the percent area protected (Table 2). Lands managed to maintain biodiversity represented between 0.6–17.0% of the area of LCCs, adding lands managed for multiple-use increased that to 1.2–62.9% (Table 2). Eight out of 16 LCCs contained ecological systems that occurred only on lands managed for multiple-use or had no permanent protection (e.g., Great Plains, North Atlantic; Figure 7). The CRI values varied across LCCs with the Eastern Tallgrass Prairie and Big Rivers having the highest value (126.4) because almost 80% of its area was converted to human use (i.e., cultivated cropland) and the Desert and Southern Rockies having the lowest (0.2) because >10% of their area contained lands managed to maintain biodiversity (Figure 8). Including lands managed for multiple-use lowered the CRI for all LCCs and increased the number of LCCs meeting the suggested Aichi Biodiversity Target of 17% target from 1 to 7 (Figure 8) [36].

**Table 2:** Total number and unique number of ecological systems as well as percent area of ecological systems on lands managed to maintain biodiversity and for multiple-use within each Landscape Conservation Cooperative (LCC) in the continental US.

Landscape Conservation Cooperative (LCC)	Number of ecological systems	Number of unique ecological systems	Percent area of lands managed to maintain biodiversity	Percent area of lands managed for multiple-use
Appalachian	103	11	3.5	8.3
California	88	2	10.7	16.3
Desert	133	2	17.0	40.0
Eastern Tallgrass Prairie & Big Rivers	75	0	1.2	1.2
Great Basin	143	1	11.2	62.9
Great Northern	143	3	14.8	39.3
Great Plains	102	1	0.6	2.5
Gulf Coast Plains & Ozarks	148	17	3.5	4.9
Gulf Coast Prairie	95	11	1.3	1.4
North Atlantic	63	5	6.6	8.7
North Pacific	123	10	15.1	25.5
Plains & Prairie Potholes	95	1	2.4	10.6
Peninsular Florida	56	18	8.8	13.1
South Atlantic	97	13	2.8	4.0
Southern Rockies	116	0	14.1	50.6
Upper Midwest & Great Lakes	60	3	5.7	8.3

See Figure for location of LCC. See Table 1 for protection status descriptions. LCCs are listed alphabetically.

## Discussion

### Protection of Ecological Systems Relative to their Occurrence in the Continental US

The existing protected areas network in the continental US would need to capture a more representative complement of ecological systems if the US aims to meet the suggested Aichi Biodiversity Target of 17% for ecologically representative terrestrial areas [36]. The 518 ecological systems mapped in the continental US are disproportionately distributed by number, size, and protection status relative to elevation and soil productivity, which translates to an uneven representation of ecological systems within the protected areas network (Figure 1A) [15], [63]. Soils with low productivity at high elevation are more likely to be found within the protected areas network; therefore ecological systems that occur in those areas are disproportionally represented in the network. Typically, low soil productivity at high elevations occurs in sparse and barren areas and these areas are well represented within the protected areas network (Figure 2) [15]. Capturing a broader range of elevation could be important to spatial patterns of biodiversity because ecological systems might shift with climate change, but the patterns of biodiversity will likely endure with geophysical features, such as elevation range [64]. How can the representation of ecological systems increase within the protected areas network of the continental US?

### Alternatives for Increasing Representation and Conservation of Ecological Systems

Many alternatives exist for conserving ecological systems and successful conservation will likely come from employing one or more of them. One approach, presented earlier in the paper, would be to replace protected areas that are minimally contributing to conservation and have a high cost associated with protecting ecological systems within a specific protected area (i.e., least cost effective) with those having greater conservation value (i.e., more cost effective) to increase the overall biodiversity protection of the entire network [49]. Applying this approach could be challenging because public support for existing protected areas may make it difficult to convince those supporters to relinquish a protected area for the benefit of the entire network [8], [65]. This approach, even though controversial because of the concept of giving up protected areas, could play a prominent role in addressing the impacts of climate change because of the potential opportunity to shift the distribution of ecological systems on current protected areas in response to shifts in temperature and precipitation [66], [67].

Protected areas have long been downgraded, downsized, delisted, and degazetted and these practices are currently widespread [68], [69]. Approximately 60 National Parks have been delisted and downgraded since the establishment of the National Park System in 1916 [68], [70], [71]. One of the major drivers of protected area degazettement, which is loss of legal protection for an entire protected area, is access to and use of natural resources (e.g., commodity extraction) [69]. The impact on biodiversity protection because of access and use of natural resources is evident in Midwestern US where a low percent area of land is managed to maintain biodiversity and many areas are mapped as human land use (Figure 4). LCC’s in the Midwest (i.e., Plains and Prairie Potholes, Great Plains, and Eastern Tallgrass Prairie and Big Rivers) have low diversity and few unique ecological systems (Figure 6B). A large percent of their area has been converted to human land use, which is reflected in high CRI values (Figure 8). To date, the ecological consequences of degazettement are unclear [69]. Both Fuller et al. [49] and Kareiva [8] believe degazettement would lead to a more dynamic and flexible approach to maintaining the current protected areas network, however it could depend on the level of systematic design used to establish the protected areas network.

Even though we did not specifically assess cost effectiveness of protected areas, our analysis could help inform the approach proposed by Fuller et al. [49]. A cost effectiveness analysis could be based on land ownership, protection status, and percent area converted to human modified systems. For example, the Great Basin LCC has potential for including some of the most cost effective protected areas because it has a low CRI value and <10% of its area is converted. There is the potential to lower its CRI value and meet the suggested 17% Aichi Biodiversity Target [36] by increasing the percent of area managed to maintain biodiversity by 60% through emphasizing protection of biodiversity (Figure 8). The Great Basin LCC also contains ecological systems that occur only on lands managed for biodiversity (Figure 7) and has a high diversity of ecological systems even though only 1 is unique (Figure 6B). Other factors beyond land ownership, protection status, and percent area converted to human modified systems could be considered in efforts to assess the cost effectiveness of protected areas, such as representation of ecological systems and transaction costs. However, our analysis could help inform a conservation strategy for the continental US if the approach described by Fuller et al. [49] were implemented.

The second alternative for improving the conservation and representation of ecological systems described previously would be to increase the size (i.e., area or number) of our existing protected areas network through acquisition for the least protected, most endangered, or high priority ecological systems [50], [51]. If a systematic approach for choosing new protected areas could increase the representation of elevation and soil productivity and thereby ecological systems then the network’s ability to respond to varying conditions and future change could be strengthened (Figure 1) [15], [63]. Our results were similar to Scott et al. [15] because we found that ecological systems at lower elevations and higher soil productivity were under-represented within the current protected areas network (Figure 1). These areas could be prioritized if acquisition of new protected areas was employed for increasing protection of ecological systems. The least protected ecological systems and potentially most endangered (see Figure 8) are within all the Level I land cover groups except sparse and barren (Figures 2, 3, and 5, Table S1) and are located mostly in the Midwestern US (Figure 4). Prioritizing acquisition of the Level I land cover groups within the Midwestern US would increase the overall representation of ecological systems in the continental US. However, the feasibility of land acquisition for conservation is continually a challenge as resources for obtaining new protected areas are dwindling and competition for undeveloped private land is limiting expansion opportunities [4], [14]. Furthermore, the support of policy makers for creating new protected areas could be perceived as ephemeral [72]. The idea of increasing the amount of protected land is attractive in part because of the perceived permanence associated with that protection. In other words, expanding the protected areas network reduces the risk of more land being converted to a state from which it might not recover (i.e., urban development), even though the immediate benefit to conservation is dependent upon management strategies employed.

A third alternative for improving the current protected areas network might be to take stock of our management within the current protected areas network and to evaluate the potential role of lands managed for multiple-use in conserving ecological systems. Our analysis found that increasing the emphasis on maintaining biodiversity on lands currently managed for multiple-use, which are permanently protected, but allow for extractive uses (e.g., mining and logging), offers an alternative for increasing the representation of ecological systems. However, much of the land managed for multiple-use has undergone ecosystem alteration and increased management or restoration may be needed to recover existing ecological systems [52]. If we increased the emphasis on maintaining biodiversity on some public and private lands managed for multiple-use, the total percent area of ecological systems protected could increase up to 39% in the continental US (lands managed to maintain biodiversity: 10%; lands managed for multiple-use: 29%). Geographically, the greatest potential for increased emphasis on maintaining biodiversity on lands managed for multiple-use is in the West, but also in the Northeast, South, and Midwest (Figure 4). To meet the suggested Aichi Biodiversity Target of 17% [36] increased emphasis on maintaining biodiversity would need to occur on 6.4% of the lands managed for multiple-use (Table S1). Even though lands managed for multiple-use occur on both public (i.e., federal, state, and local government) and private (i.e., non-governmental organization) lands, the potential for conservation efforts to increase the protection of ecological systems on public lands is greater (i.e., quantitatively and geographically) (Figure 5).

To protect a broad representation of ecological systems within the continental US, opportunities within public land management agencies fall largely on lands managed by BLM and USFS (Figure 5). Both manage lands that maintain biodiversity, but the majority of the lands they manage are for multiple-use (Figure 5). However, if the US is to become less dependent on foreign energy sources and meet its own resource needs within its boundaries, then shifting management focus on even a small portion of lands currently managed for multiple-use could become a public lands dilemma. Lands managed for multiple-use provide multiple public benefits, including domestic energy production. [17], [73], [74].

In addition to the lands BLM manages for multiple-use, it has also designated 11 million hectares to the National Landscape Conservation System (NLCS), which is a network of conservation areas specifically aimed at conserving biodiversity [75]. The USFS manages over 17 million hectares of land managed to maintain biodiversity, which is more than USFWS, NPS, and other federal land management agencies combined (Figure 5). With BLM and USFS managing millions of hectares of land for maintaining biodiversity, their role in protecting ecological systems is well established, and there may be potential to expand the protection and representation of ecological systems, for example, through the expansion of NLCS. In the past, administrative jurisdictional land transfers have occurred between land management agencies (e.g., BLM, USFWS, NPS, and USFS) [76]–[78]. Some of these land transfers have led to more emphasis on maintaining biodiversity.

### Landscape Conservation Cooperatives Setting Priorities for Conservation of Ecological Systems

The framework and partnerships of the LCCs informs conservation at the landscape level, which will be needed to implement conservation across jurisdictional boundaries. Our analysis indicates that ecological systems in the East are less redundant and at more risk of conversion than those in the West (Figures 6 and 8). Because of this East-West dichotomy, increased conservation on some public and private lands may be important to the representation of ecological systems in the West, whereas increased public-private partnerships may play an important role in the East to increase the representation of ecological systems (Figures 4, 5, 6, 7, 8).

Our research results highlighting low redundancy and unique ecological systems corroborate results from other studies [13], [18]. In particular, the eastern US was identified as an ecoregion with high threats and irreplaceability value with regards to identifying conservation priorities [13], [18]. For example, the Gulf Coast Plain and Ozarks LCC in southeastern US has high diversity and uniqueness, but low redundancy and a high conservation risk index (Figures 6 and 8). Within this LCC, there are few opportunities for increasing the representation of ecological systems on lands managed for multiple-use (Table 2, percent protected changes from 3.5% to 4.9%). An initial practical approach for conservation of ecological systems in this LCC, which contains many diverse and unique ecological systems, would be to engage both public and private conservation partners. In this case, our research results could serve as a catalyst for building public and private conservation partnerships. The larger scale perspective of LCCs provides a unique forum that previously did not exist for putting nationwide conservation planning at a scale that allows strategic emphasis on ecological systems that are in most need of added representation and protection.

There are numerous benefits to exploring alternatives for increasing the conservation and representation of ecological systems in the protected areas network. First, we can increase the number and area of ecological systems protected. Ecological systems represent a range of the habitats upon which many species rely; therefore we are increasing the protection of numerous species, including threatened, endangered, and species of concern. Second, we can increase the adaptability of ecological systems and the protected areas network to climate change impacts [79]. A wider range of environmental variables will enable ecological systems and the vertebrate species that rely on them to have room to shift their ranges in response to changes in climate. Third, we can increase the buffer area for all ecological systems and thereby reduce edge effects and increase the integrity of existing ecological systems. Lastly, we are more likely to capture the ecological processes that drive the pattern of ecological systems that we observe and allow for a more fully functional and robust protected areas network.

The current protected areas network for the continental US does not capture the full range of ecological systems or geophysical features (i.e., elevation and soil productivity). As a consequence, the species that rely on these ecological systems and geophysical features have fewer opportunities to adjust to changing environmental conditions. We have not assessed the pros and cons of using our alternatives for increasing the representation of ecological systems, but rather we have presented them as possibilities that may be considered and evaluated as decisions are made to conserve biodiversity. Each alternative may increase the representation of ecological systems, which can lead to protecting and securing habitat across a broader range of ecological, geographical, and geophysical occurrence of species. And may provide the greatest opportunity for evolutionary processes to persist regardless of imminent changes in the near, intermediate, and long term.

## Supporting Information

Table S1 **Area (ha) and percent area of ecological systems by protection status nested into Level I and II land cover groups** **[23]**, **[24]** **.** All 5 Level I groups, 37 Level II groups, and 518 ecological systems are listed. See Table 1 for protection status descriptions. Only non-modified, non-aquatic ecological systems are included (n = 518).
(XLSX)

## References

[1] Miller KR (1982) Parks and protected areas: considerations for the future. Ambio 11: 315–317.

[2] Pressey RL (1994) Ad hoc reservations: forward and backward steps in developing representative reserve systems? Conserv Biol 8: 662–668.

[3] Margules CR, Pressey RL (2000) Systematic conservation planning. Nature 405: 243–253. 10821285 10.1038/35012251

[4] Fairfax SK, Gwin L, King MA, Raymond L, Watt LA (2005) Buying nature: The limits of land acquisition as a conservation strategy, 1780–2004. Cambridge: The MIT Press. 357 p.

[5] Pressey RL, Humphries CJ, Margules CR, Vane-Wright RI, Williams PH (1993) Beyond opportunism: key principles for systematic reserve selection. Trends Ecol Evol 8: 124–128. 21236127 10.1016/0169-5347(93)90023-I

[6] Ando A, Camm J, Polasky S, Solow A (1998) Species distributions, land values, and efficient conservation. Science 279: 2126–2128. 9516117 10.1126/science.279.5359.2126

[7] van Jaarsveld AS, Freitag S, Chown SL, Muller C, Koch S, et al (1998) Biodiversity assessment and conservation strategies. Science 279: 2106–2108. 9516111 10.1126/science.279.5359.2106

[8] Kareiva P (2010) Trade-in to trade-up. Nature 466: 322–323. 20631786 10.1038/466322a

[9] Harrison J, Miller K, McNeely J (1982) The world coverage of protected areas: development goals and environmental needs. Ambio 11: 238–245.

[10] Ehrlich PR, Wilson EO (1991) Biodiversity studies: science and policy. Science 253: 758–762. 17835492 10.1126/science.253.5021.758

[11] Scott JM, Davis F, Csuti B, Noss R, Butterfield B, et al (1993) Gap analysis: a geographic approach to protection of biological diversity. Wildlife Monographs 123: 1–41.

[12] Chape S, Blyth S, Fish L, Fox P, Spalding M (2003) 2003 United Nations list of protected areas. Available: http://www.unep.org/pdf/un-list-protected-areas.pdf. Access 29 February 2012.

[13] Rodrigues ASL, Akçakaya HR, Andelman SJ, Bakarr MI, Boitani L, et al (2004) Global gap analysis: priority regions for expanding the global protected-area network. Bioscience 54: 1092–1100.

[14] McDonald RI (2009) The promise and pitfalls of systematic conservation planning. Proc Natl Acad Sci U S A 106: 15101–15102. 19805246 10.1073/pnas.0908125106 PMC2741211

[15] Scott JM, Davis FW, McGhee RG, Wright RG, Groves C, et al (2001) Nature preserves: do they capture the full range of America’s biological diversity? Ecol Appl 11: 999–1007.

[16] Baron JS, Griffith B, Joyce LA, Kareiva P, Keller BD, et al. (2008) Preliminary review of adaptation options for climate-sensitive ecosystems and resources. Available: http://library.globalchange.gov/products/assessments/sap-4-4-preliminary-review-of-adaptation-options-for-climate-sensitive-ecosystems-and-resources. Accessed 29 February 2012.

[17] Glicksman RL (2008) Ecosystem resilience to disruptions linked to global climate change: An adaptive approach to federal land management. Neb Law Rev 87: 833–892.

[18] Brooks TM, Bakarr MI, Boucher T, Da Fonseca GAB, Hilton-Taylor C, et al (2004) Coverage provided by the global protected-area system: is it enough? Bioscience 54: 1081–1091.

[19] Estes JE, Mooneyhan DW (1994) Of maps and myths. Photogramm Eng Remote Sensing 60: 517–524.

[20] Dietz RW, Czech B (2005) Conservation deficits for the continental United States: an ecosystem gap analysis. Conserv Biol 19: 1478–1487.

[21] Noss RF, Cooperrider AY (1994) Saving Nature’s legacy. Washington DC: Island Press. 416 p.

[22] The President’s Council of Advisors on Science and Technology (PCAST) (2011) Sustaining environmental capital: protecting society and the economy. Available: http://www.whitehouse.gov/administration/eop/ostp/pcast/docsreports. Accessed 2011 July 29.

[23] US Geological Survey, Gap Analysis Program (USGS-GAP) (2010) National GAP Land Cover, Version 1. Available: http://gapanalysis.usgs.gov. Accessed 29 July 2011.

[24] US Geological Survey, Gap Analysis Program (USGS-GAP) (2010) Protected Areas Database of the United States, Version 1.0. Available: http://gapanalysis.usgs.gov. Accessed 2011 July 29.

[25] Comer P, Faber-Langendoen D, Evans R, Gawler S, Josse C, et al. (2003) Ecological systems of the United States: a working classification of US terrestrial systems. Available: http://www.natureserve.org/library/usEcologicalsystems.pdf. Accessed 29 July 2011.

[26] Redford KH, Richter BD (1999) Conservation of biodiversity in a world of use. Conserv Biol 13: 1246–1256.

[27] Sanderson EW, Jaiteh M, Levy MA, Redford KH, Wannebo AV, et al (2002) The human footprint and the last of the wild. Bioscience 52: 891–904.

[28] Hobbs RJ, Arico S, Aronson J, Baron JS, Bridgewater P, et al (2006) Novel ecosystems: theoretical and management aspects of the new ecological world order. Global Ecol Biogeogr 15: 1–7.

[29] Sodhi NS, Butler R, Laurance WF, Gibson L (2011) Conservation successes at micro-, meso-, and macroscales. Trends Ecol Evol 26: 585–594. 21824677 10.1016/j.tree.2011.07.002

[30] Noss RF, LaRoe III ET, Scott JM (1995) Endangered ecosystems of the United States: A preliminary assessment of loss and degradation. Available: http://biology.usgs.gov/pubs/ecosys.htm. Accessed 2010 July 22.

[31] Bunce RGH, Bogers MMB, Evans D, Halada L, Jongman RHG, et al. (2012). The significance of habitats as indicators of biodiversity and their links to species. Ecol Indic http://dx.doi.org/10.1016/j.ecolind.2012.07.014. Accessed 2012 August 31.

[32] Dudley N (2008) Guidelines for applying protected area management categories. Available: http://data.iucn.org/dbtw-wpd/edocs/paps-016.pdf. Accessed 2012 February 29.

[33] Noss RF (1990) Indicators for monitoring biodiversity: a hierarchical approach. Conserv Biol 4: 355–364.

[34] Tear TH, Kareiva P, Angermeier PL, Comer P, Czech B, et al (2005) How much is enough? The recurrent problem of setting measurable objectives in conservation. Bioscience 55: 835–849.

[35] Svancara LK, Brannon R, Scott JM, Groves CR, Noss RF, et al (2005) Policy-driven versus evidence-based conservation: a review of political targets and biological needs. Bioscience 55: 989–995.

[36] Convention on Biological Diversity Strategic Plan for Biodiversity 2011–2020 including Aichi Biodiversity Targets. Available:.Accessed 2012 February 29.

[37] Shaffer ML, Stein BA (2000) Safeguarding our precious heritage. In: Stein BA, Kutner LS, Adams JS, editors. Precious heritage: the status of biodiversity in the United States. New York: Oxford University Press. 301–321.

[38] Groves CR (2003) Drafting a conservation blueprint: a practioner’s guide to planning for biodiversity. Washington DC: Island Press. 458 p.

[39] Scott D, Malcolm JR, Lemieux C (2002) Climate change and modelled biome representation in Canada’s national park system: implications for system planning and park mandates. Global Ecol Biogeogr 11: 475–484.

[40] Lemieux CJ, Scott DJ (2005) Climate change, biodiversity conservation and protected area planning in Canada. Can Geogr 49: 384–399.

[41] Lemieux CJ, Beechey TJ, Gray PA (2011) Prospects for Canada’s protected areas in an era of rapid climate change. Land use policy Available: doi:10.1016/j.landusepol.2011.03.008. Accessed 2011 April 29.

[42] Shelford VE (1926) Naturalist’s guide to the Americas. Baltimore: The Williams and Wilkins Company. 761 p.

[43] Crumpacker DW, Hodge SW, Friedley D, Gregg WP (1988) A preliminary assessment of the status of major terrestrial and wetland ecosystems on Federal and Indian lands in the United States. Conserv Biol 2: 103–115.

[44] Caicco SL, Scott JM, Butterfield B, Csuti B (1995) A gap analysis of the management status of the vegetation of Idaho (USA). Conserv Biol 9: 498–511.

[45] Davis FW, Stine PA, Stoms DM, Borchert MI, Hollander AD (1995) Gap analysis of the actual vegetation of California 1. The Southwestern Region. Madroño 42: 40–78.

[46] Stoms DM, Davis FW, Driese KL, Cassidy KM, Murray MP (1998) Gap analysis of the vegetation of the intermountain semi-desert ecoregion. Great Basin Nat 58: 199–216.

[47] Scott JM, Murray M, Wright RG, Csuti B, Morgan P, et al (2001) Representation of natural vegetation in protected areas: capturing the geographic range. Biodivers Conserv 10: 1297–1301.

[48] Wright RG, Scott JM, Mann S, Murray M (2001) Identifying unprotected and potentially at risk plant communities in the western USA. Biol Conserv 98: 97–106.

[49] Fuller RA, McDonald-Madden E, Wilson KA, Carwardine J, Grantham HS, et al (2010) Replacing underperforming protected areas achieves better conservation outcomes. Nature 466: 365–367. 20592729 10.1038/nature09180

[50] Langhammer PF, Bakarr MI, Bennun LA, Brooks TM, Clay RP, et al.. (2007) Identification and gap analysis of key biodiversity areas: targets for comprehensive protected area systems. Available: data.iucn.org/dbtw-wpd/edocs/pag-015.pdf. Accessed 2012 February 29.

[51] Kark S, Levin N, Grantham HS, Possingham HP (2009) Between-country collaboration and consideration of costs increase conservation planning efficiency in the Mediterranean Basin. Proc Natl Acad Sci U S A 106: 15368–15373. 19717457 10.1073/pnas.0901001106 PMC2741257

[52] Swaty R, Blankenship K, Hagen S, Fargione J, Smith J, et al (2011) Accounting for ecosystem alteration doubles estimates of conservation risk in the conterminous United States. PLoS ONE 6: 1–10 DOI:10.1371/journal.pone.0023002. 10.1371/journal.pone.0023002 PMC3151280 21850248

[53] Millard MJ, Czarnecki CA, Morton JM, Brandt LA, Shipley FS, et al (2012) A national geographic framework for guiding conservation on a landscape scale. Journal of Fish and Wildlife Management 3: 175–183.

[54] Lowry Jr JH, Ramsey RD, Boykin K, Bradford D, Comer P, et al. (2007) Land cover classification and mapping. In: Prior-Magee JS, et al., editors. Southwest Regional Gap Analysis Final Report. Available: http://fws-nmcfwru.nmsu.edu/swregap/report/swregap%20final%20report.pdf. Accessed 2011 November 16.

[55] Zhu Z, Vogelmann J, Ohlen D, Kost J, Chen X, et al. (2006) Mapping existing vegetation composition and structure for the LANDFIRE Prototype Project. In: Rollins MG, Frame CK, editors. The LANDFIRE prototype project: nationally consistent and locally relevant geospatial data for wildland fire management Available: http://www.treesearch.fs.fed.us/pubs/24700. Accessed 2011 January 18.

[56] Rollins MG (2009) LANDFIRE: a nationally consistent vegetation, wildland fire, and fuel assessment. International Journal of Wildland Fire 18: 235–249.

[57] Sanborn (2006) GAP zone 1 vegetation mapping final report. Available: http://gap.uidaho.edu. Accessed 2011 July 29.

[58] Lowry J, Ramsey RD, Thomas K, Schrupp D, Sajwaj T, et al (2007) Mapping moderate-scale land-cover over very large geographic areas within a collaborative framework: a case study of the Southwest Regional Gap Analysis Project (SWReGAP). Remote Sens Environ 108: 59–73.

[59] Crist PJ, Prior-Magee JS, Thompson BC (1996) Land management status categorization in gap analysis: a potential enhancement. In Brackney ES, Jennings MD, editors. Gap Analysis Bulletin 5. Available: http://www.gap.uidaho.edu/bulletins/5/LMSCiGA.html. Accessed 2010 March 29.

[60] U. S. Geological Survey (2006) National elevation dataset. Available: http://ned.usgs.gov/. Accessed 2011 July 29.

[61] Hazen HD, Anthamatten PJ (2004) Representation of ecological regions by protected areas at the global scale. Physical Geography 25: 499–512.

[62] Hoekstra JM, Boucher TM, Ricketts TH, Roberts C (2005) Confronting a biome crisis: global disparities of habitat loss and protection. Ecol Lett 8: 23–29.

[63] Groves CR, Kutner LS, Stoms DM, Murray MP, Scott JM, et al.. (2000) Owning up to our responsibilities: who owns land important for biodiversity? In: Stein BA, Kutner LS, Adams JS, editors. Precious heritage: The status of biodiversity in the United States. New York: Oxford University Press. 399 p.

[64] Andersen MG, Ferree CE (2010) Conserving the stage: climate change and the geophysical underpinnings of species diversity. PLoS ONE 5: 1–10 DOI:10.737/journal.pone.0011554. 10.1371/journal.pone.0011554 PMC2904386 20644646

[65] Tversky A, Kahneman D (1974) Judgment under uncertainty: heuristics and biases. Science 185: 1124–1131. 17835457 10.1126/science.185.4157.1124

[66] Parmesan C (2006) Ecological and evolutionary responses to recent climate change. Annu Rev Ecol Evol Syst 37: 637–669.

[67] Parmesan C, Yohe G (2003) A globally coherent fingerprint of climate change impacts across natural systems. Nature 421: 37–42. 12511946 10.1038/nature01286

[68] Retti DR (1995) Our National Park System: caring for America’s greatest natural and historic treasures. Urbana: University of Illinois Press. 293 p.

[69] Mascia MB, Pailler S (2011) Protected area downgrading, downsizing, and degazettement (PADDD) and its conservation implications. Conservation Letters 4: 9–20.

[70] Hogenauer AK (1991) Gone, but not forgotten: The delisted units of the US National Park System. The George Wright Forum 7: 2–19.

[71] Hogenauer AK (1991) An update to “Gone, but not forgotten: The delisted units of the US National Park System. The George Wright Forum. 8: 26–28.

[72] US Department of Interior and US Department of Agriculture (2005) National Land Acquisition Plan. Available: http://www.fs.fed.us/land/staff/LWCF/Final%20DOI-USDA%20Land%20Acquisition%20Report%20to%20Congress.pdf. Accessed 2012 September 5.

[73] Loomis JB (1993) Integrated public lands management. New York: Columbia University Press 474 p.

[74] Thomas JW, Sienkiewicz A (2005) The relationship between science and democracy: public land policies, regulation, and management. Public Land and Resources Law Review 26: 39–69.

[75] Darst CR, Huffman KA, Jarvis J (2009) Conservation significance of America’s newest system of protected areas: National Landscape Conservation System. Natural Areas Journal 29: 224–254.

[76] Towns E, Cook JE (1998) USDA, Forest Service, USDI, National Park Service: Notice of Transfer of Administrative Jurisdiction, Coconino National Forest and Walnut Canyon National Monument. Available: http://www.gpo.gov/fdsys/pkg/FR-1998-08-25/pdf/98-22723.pdf. Accessed 2012 August 31.

[77] Stobaugh J (2003) Notice of Proposed Withdrawal and Opportunity for Public Meeting: Nevada. Available: http://www.gpo.gov/fdsys/pkg/FR-2003-07-09/pdf/03-17392.pdf. Accessed 2012 August 31.

[78] Allred CS (2007) Public Land Order No. 7675: Transfer of Administrative Jurisdiction, Petrified Forest National Park Expansion, Arizona. Available: http://www.gpo.gov/fdsys/pkg/FR-2007-05-18/pdf/E7-9586.pdf. Accessed 2012 August 31.

[79] Kujala H, Araújo MB, Thuiller W, Cabeza M (2011) Misleading results from conventional gap analysis – messages from the warming north. Biol Conserv 144: 2450–2458.

